# Novel Effects of Combination Therapy Through Inhibition of Caspase-1/Gasdermin D Induced-Pyroptosis in Lupus Nephritis

**DOI:** 10.3389/fimmu.2021.720877

**Published:** 2021-11-19

**Authors:** Heng Cao, Junyu Liang, Jing Liu, Ye He, Yini Ke, Yiduo Sun, Song Jiang, Jin Lin

**Affiliations:** ^1^ Department of Rheumatology, The First Affiliated Hospital, Zhejiang University School of Medicine, Hangzhou, China; ^2^ National Clinical Research Center of Kidney Diseases, Jinling Clinical Medical College of Nanjing Medical University, Nanjing, China

**Keywords:** lupus nephritis, pyroptosis, caspase-1, gasdermin D, therapy

## Abstract

**Objectives:**

Combination therapy with mycophenolate mofetil, tacrolimus and steroids are effective in achieving complete remission in lupus nephritis (LN). Combination therapy uniquely downregulated caspase-1 compared with monotherapies, which can cleave gasdermin D (GSDMD) and was recently identified as the pyroptosis executioner. We therefore investigated whether combination therapy enabled the suppression of caspase-1/GSDMD-mediated pyroptosis in LN.

**Methods:**

Expression and activation of GSDMD were detected in kidney specimens of the human and mouse with LN using immunohistochemical staining and immunoblotting. Primary podocytes isolated from MRL/lpr mice were incubated with LPS+ATP, and pretreated with monotherapy or combination therapy. Inhibition of caspase-1/GSDMD-induced pyroptosis by combination therapy were assessed in MRL/lpr mice and human specimens. Pyroptosis was examined using a FAM caspase-1 kit and flow cytometry. The correlation between pyroptosis in peripheral blood and the systemic lupus erythematosus disease activity index (SLEDAI) was analyzed.

**Results:**

Kidney tissue specimens from LN patients and mice exhibited greatly increased expression levels and cleavage of GSDMD. In cultured podocytes, combination treatment significantly suppressed the activation of NLRP3 and caspase-1 and reduced GSDMD N-terminal levels. Combination therapy repressed disease progression through inhibition of caspase-1/GSDMD-mediated pyroptosis in both humans and MRL/lpr mice. Caspase-1/PI positive cell numbers in peripheral blood were positively correlated with SLE-DAI. LN patients with complete remission and partial remission had remarkably reduced caspase-1/PI positive cell numbers compared to baseline. Ac-FLTD-CMK, a GSDMD-derived inhibitor, prevented the development of LN.

**Conclusion:**

Combination therapy suppressed caspase-1/GSDMD-mediated pyroptosis *in vitro* and *in vivo* and reduced disease progression.

## Introduction

Lupus nephritis (LN) is kidney inflammation arising as a consequence of the autoimmune disease systemic lupus erythematous (SLE). LN is a major manifestation in patients who suffer from SLE. About 60% of patients develop this condition, with approximately 20% progressing to end-stage kidney disease (ESRD) (1). Combination therapy with mycophenolate mofetil (MMF), calcineurin inhibitors (CNI) and steroids produces complementary effects and also minimizes toxicity (2). This therapy is recommended as the initial treatment for LN and produces better efficacy and more complete remission compared with intravenous cyclophosphamide (IVCY) and steroids (3–5).

To investigate the molecular and cellular mechanisms of combination therapy, comprehensive transcriptomic analysis was employed in a murine model of LN. MRL/lpr mice were given alone MMF, tacrolimus or prednisone as monotherapy, or triple drug combinations. Profiles of the expression of genes in the kidneys of mice with LN revealed potential mechanisms that might account for the additive and synergistic effects of combination therapy compared with monotherapies. One of 45 downregulated genes that is uniquely modulated by combination therapy is caspase-1 (6). It is activated by inflammasomes, which are multiprotein oligomers comprised of NLRP3, pro-caspase-1 and apoptosis-associated speck like protein (ASC), with an integrated caspase affinity domain. The NLRP3/ASC/caspase-1 inflammasome has been recognized as a key contributor to the pathogenesis of LN. The NLRP3 inflammasome drives autoinflammatory disorders by activating caspase-1, initiating the generation of pro-inflammatory cytokines e.g. IL-1β, which is known to trigger podocyte dysfunction in LN (7–9).

Active caspase-1 cleaves gasdermin D (GSDMD) within the linkage of its N- and C-terminal domains. Then, the N-terminal domain facilitates pore formation in the cell membrane, stimulates cell pyroptosis and cytokine IL-1β and IL-18 production (10). Emerging studies have shown that GSDMD-mediated pyroptosis participates in the progression of diseases including autoimmune encephalomyelitis (11), multiple sclerosis (12), alcoholic hepatitis (13) and inflammatory bowel disease (14). In addition, genetic or pharmacological inhibition of caspase-1/GSDMD has been demonstrated to protect the kidneys from injury in murine models of diabetic kidney disease (15, 16) and also after acute kidney injury (17–19). However, pyroptosis in LN is still poorly defined to date (20). We hypothesized that combination therapy conferred beneficial effects in LN by inhibiting pyroptosis induced by caspase-1/GSDMD.

In the present study, the activation of caspase-1 and GSDMD in kidney tissues was investigated as well as the effects of combination therapy on caspase-1/GSDMD-dependent pyroptosis inhibition in MRL/lpr mice and in patients with LN. Potential correlations of pyroptosis with the SLE disease activity index (SLEDAI) and the treatment responses were also assessed.

## Materials and Methods

### Study Population

The study cohort was comprised of patients diagnosed by biopsy with LN ≤ 6 months before enrollment. The patients age range was 18–65 years and all met the classification criteria of the American College of Rheumatology for SLE (21, 22) and the International Society of Nephrology/Renal Pathology Society (2003). Patients were recruited from the Zhejiang University School of Medicine and Nanjing University School of Medicine who had proteinuria (≥ 1.5 g/d) and serum creatinine concentrations ≤ 265.2 μmol/L. The exclusion criteria included: a patient had previously been treated with high doses of methylprednisolone; biologics (targeting specific pathogenic molecules) or combination therapy; current plasmapheresis or treatment with intravenous globulin within 12 weeks of screening; liver functions that were abnormal; current active infections; and a malignant tumor within 4 weeks of commencement of the study. LN patients received pulse therapy with methylprednisolone for 3 days at a dose of 0.5 g/d. Once methylprednisolone pulse therapy was completed, the combination group with prednisone (0.6 mg/kg/d), MMF (0.5 g, two doses/d) and tacrolimus (2 mg, two doses/d) were given for a total of 4 weeks. Then the doses of prednisone, which were still combined with MMF (0.5 g, two doses/d) and tacrolimus (2 mg, two doses/d), were reduced by 5 mg/d every 2 weeks until a 10 mg/d maintenance dose was achieved (4). Complete remission (CR) was taken as a 24-h urinary protein concentration ≤ 0.4 g, the absence of active urine sediments, a serum albumin concentration ≥ 35 g/L, and normal creatinine (23). Partial remission (PR) was taken as a ≥ 50% decrease in the 24-h urinary protein concentration to ≤ 3.5 g, a serum albumin concentration of ≥ 30 g/L, and a normal or ≤ 25% increase in the serum creatinine concentration from baseline. Our Institutional Review Board approved the study (No. IIT20200410A) and all patients provided written consent prior to enrollment.

### Animal Models

Female MRL/mpj-Fas^lpr^ (MRL/lpr) mice were supplied by the SLAC Laboratory Animal Co. Ltd (Shanghai, China), all mice were randomly divided with the help of random number generator, 5 mice in each group and each cage. They were treated at age 8 weeks orally, with a combination of 0.5 mg/kg tacrolimus (MCE HY-13756), 50 mg/kg MMF (MCE HY-B0199) and 1 mg/kg prednisone (MCE HY-B0214) or vehicle daily for a total 8 weeks as previously described (6). Ac-FLTD-CMK was synthesized by MCE and 10 mg/kg was injected i.p. daily. All treatment were blinded to the participants responsible for the following detection. The urine albumin-to-creatinine ratio (UACR) was determined using Albuwell M and the Creatinine Companion (Exocell, Phil., US). Kidney specimens were fixed in 4% paraformaldehyde prior to histological and Immunohistochemical staining and kidney cortices extracted for immunoblotting analysis. Mice use was approved by our Institutional Animal Care and Use Committee (No. 2019-883).

### Mice Glomeruli Isolation and Podocyte Culture

Glomeruli were purified from the kidneys of 12-week-old female C57 and MRL/lpr mice as described before (24). Anesthetized mice were perfused with 37°C preheated magnetic beads. The kidneys were removed and minced on ice into 1 mm^3^. Then the renal tissues were digested at 37°C with 1 mg/ml collagenase and 100 U/ml DNase I for 30 minutes, then filtered twice with 100-µm Falcon cell strainers (431752, BD). After several washes with 4°C HBSS solution (H1025, Solarbio) and gentle centrifugation at 200g for 5 minutes, glomeruli containing beads were collected by using the magnetic stand (HY-K0200, MCE) and washed with HBSS solution three times. Isolated glomeruli were cultured on type I collagen (C3867, Sigma-Aldrich) coated cultured dishes in 5% CO_2_ at 37°C according to the method described by Jeffrey (25). Passage 2 podocytes were used *in vitro* experiment.

### Pyroptosis Induction, Cytotoxicity and IL-1β Secretion Detection

Podocytes from MRL/lpr mice were primed with 1mg/ml LPS (tlrl-pb5lps, *In vivo*gen) resolved in Opti-MEM for 4h, then followed by 5mM ATP (tlrl-atpl, *In vivo*gen) stimulation. Podocytes from C57 mice were severed as normal control. Monotherapies tacrolimus (10µM), MMF (1µM) and prednisone (10µM), or combined therapy was pre-incubated with the cells for 1 h before LPS priming. The cytotoxicity after stimulation was determined by using cytotoxicity detection kit (11644793001, Roche) according to the manufacturer’s instructions. The supernatant after stimulation were collected and measured using the IL-1β mouse ELISA Kit (BMS6002, Invitrogen) according to the manufacturer’s instructions.

### Antibodies and Reagents

Caspase-1 (sc-398715, Santa Cruz Biotech), GSDMDC1 (sc-81868, Santa Cruz Biotech) levels were assessed as per the manufacturers’ instructions. Pyroptosis in peripheral blood was measured using FAM FLICA^TM^ Caspase-1 Kit (Bio Rad, USA) by flow cytometry.

### Statistical Analysis

Statistical analyses were performed using GraphPad Prism (vers. 8.0) and the data are given as means ± SD. Two group comparisons were conducted using Student’s *t*-test and correlation analyses using Pearson’s correlation. Two-tailed *p*<0.05 was considered statistically significant.

## Results

### GSDMD-Mediated Pyroptosis Is Activated in Kidneys Affected by LN

**Figure 1:**
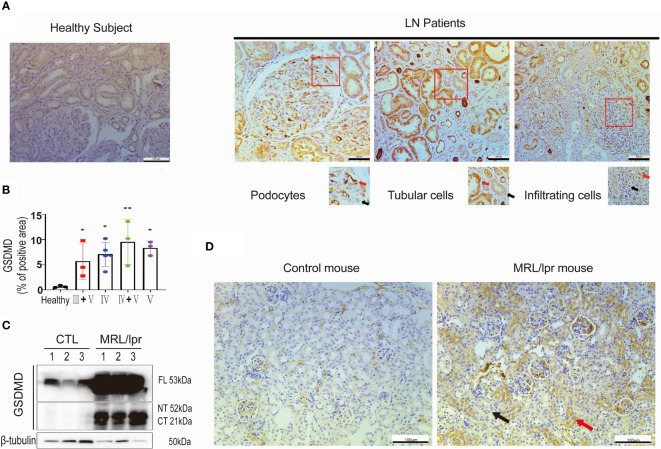
GSDMD is strongly elevated and cleaved in the kidneys of patients and mice affected by LN. **(A)** Immunochemical staining of GSDMD in renal specimens taken from healthy subjects and LN patients, positive (Red arrows) and negative staining (Black arrows) were enlarged. **(B)** Quantification of GSDMD-positive areas is shown in type III+V, IV, IV+V, V LN patients and heathy subjects. **(C)** Immunoblot analysis of full-length, cleaved N-terminal domain and C-terminal domain GSDMD in renal cortex from three pairs of control and MRL/lpr mice. **(D)** Immunochemical staining of GSDMD in kidney specimens taken from controls and MRL/lpr mice. ^*^ *p* < 0.05, ^**^ *p* < 0.01. LN, lupus nephritis; FL, full-length; NT, N-terminal domain; CT, C-terminal domain.

To investigate whether GSDMD contributed to the pathogenesis of LN, we first tested the protein expression level of GSDMD in kidney specimens taken from LN patients. Immunohistochemical analysis revealed remarkable increased expression levels of GSDMD in kidney biopsy specimens taken from LN patients with type IV, V, III + V and IV + V, and compared to healthy subjects. GSDMD was universally expressed in glomerular podocytes, tubular cells and infiltrated interstitial cells in LN patients. The area with positive (Red arrows) and negative staining (Black arrows) were enlarged (**Figure 1A** **)**. The percentage of GSDMD-positive area in the kidney was then estimated (**Figure 1B** **)**. Similar results were found in kidneys of MRL/lpr mice and wild-type controls. Immunoblotting analysis confirmed greatly increased expression and cleavage of GSDMD in kidney specimens of MRL/lpr mice **(** **Figure 1C** **)**. The GSDMD in glomerular podocytes, tubular cells and infiltrated interstitial cells was elevated in MRL/lpr mice compared to control mice **(** **Figure 1D** **)**. These data suggested that GSDMD-mediated pyroptosis was activated in renal tissue affected by LN.

### Combination Treatment Suppresses GSDMD-Mediated Pyroptosis Activation in Primary Podocytes *In Vitro*

**Figure 2:**
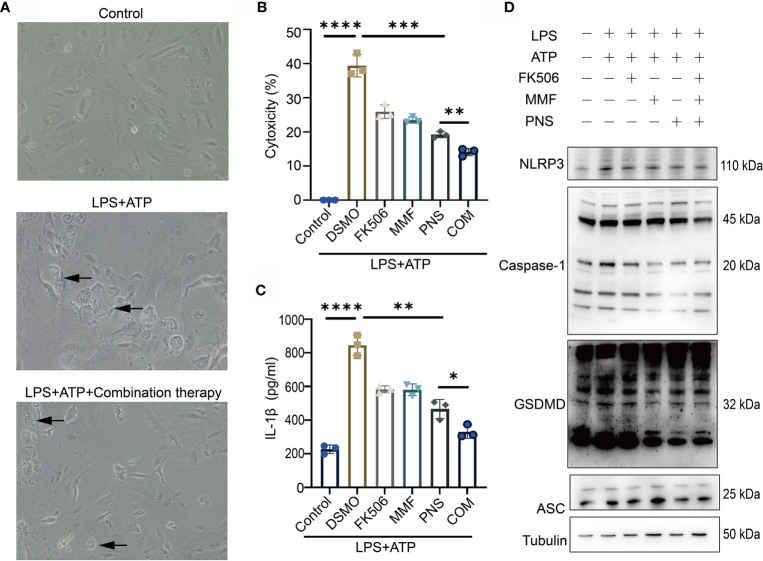
Combination treatment ameliorates pyroptosis in podocytes *in vitro*. **(A)** Pyroptotic features were exhibited in cultured primary podocytes after incubated with LPS+ATP, and pretreated with combination treatment. Arrows indicated cells exhibiting pyroptotic-like features. **(B)** Supernatants from challenged cells were analyzed for cell death, as measured by LDH secretion. **(C)** Supernatants from the challenged cells were analyzed for IL-1β release. **(D)** Immunoblotting analysis of NLRP3, caspase-1, GSDMD-NT and ASC expression after tacrolimus, MMF, prednisone or combination treatment after NLRP3 inflammasome activation. ^*^ *p* < 0.05; ^**^ *p* < 0.01; ^***^ *p* < 0.001; ^****^ *p* < 0.0001. LDH, lactate dehydrogenase; DSMO, Dimethyl sulfoxide, FK506, tacrolimus; MMF, mycophenolate mofetil; PNS, prednisone; COM, combination therapy; ASC, apoptosis-associated speck like protein.

To investigate the inhibitory effects of combination therapy on pyroptosis, we applied LPS+ATP stimulation method to induce pyroptosis in podocytes from MRL/lpr mice, taking podocytes from wild type C57 mice as the normal control. Podocytes from MRL/lpr mice were primed for 4 h with LPS and subsequently treated for 4 h with ATP, a commonly used stimulus for GSDMD-mediated pyroptosis activation (26). Comparing with normal control, podocytes afterwards exhibited pyroptotic morphological features such as cell swelling and bubbling. Pretreatment with combination therapy significantly ameliorated pyroptotic morphological features and cytotoxicity **(** **Figure 2A** **)**. LPS+ATP clearly induced podocytes cytotoxicity. Comparably, combined therapy significantly decreased the cytotoxicity and IL-1β secretion in supernatant induced by LPS+ATP stimulation **(** **Figures 2B, C** **)**. To verify further the activation of GSDMD-mediated pyroptosis, we analyzed canonical inflammasomes and GSDMD activation using immunoblotting. Consistent with pyroptotic morphological changes and cytotoxicity, LPS+ATP significantly activated NLRP3, caspase-1, and cleaved GSDMD in podocytes **(** **Figure 2D** **)**. Combination treatment markedly suppressed the activation of NLRP3 and caspase-1, and reduced the level of GSDMD N-terminal. Notably, monotherapies with tacrolimus, MMF or prednisone suppressed LPS+ATP stimulated cell cytotoxicity and IL-1β secretion, but combination therapy showed a more significant inhibitory effect on Caspase-1 and GSDMD. These results provided evidence which supported the view that combination treatment ameliorated GSDMD-mediated pyroptosis by suppressing the activation of NLRP3 and caspase-1.

### Combination Treatment Suppressed Caspase-1/GSDMD-Induced Pyroptosis in MRL/lpr Mice

Eight-week-old MRL/lpr mice were given a combination of MMF, tacrolimus and prednisone or vehicle control. Consistent with a previous study (6), after treatment for 8 weeks, no difference was found in mortality or body weight between the vehicle and combination treatment groups. In contrast to the vehicle group, combination therapy was very effective in preventing proteinuria, glomerulosclerosis and renal interstitial immune cells infiltration (**Supplementary Figure 1**).

**Figure 3:**
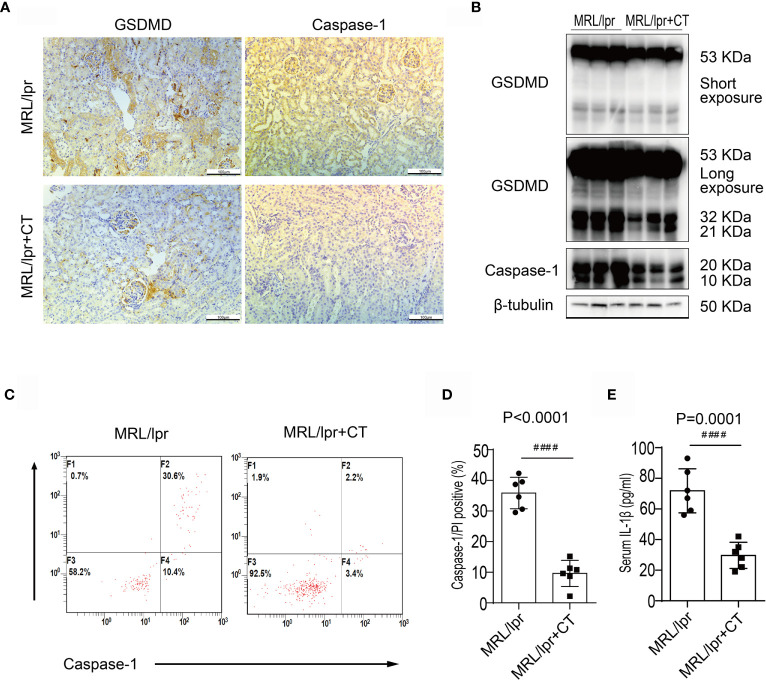
Combination treatment attenuated caspase-1/GSDMD-mediated pyroptosis in MRL/lpr mice. **(A)** Immunochemical staining of GSDMD and caspase-1 in MRL/lpr mice kidney specimens. Compared to the vehicle control, caspase-1 and GSDMD expression was markedly inhibited in the combination treatment group. **(B)** Immunoblot analysis of full-length and cleaved GSDMD and caspase-1 in renal cortex specimens of MRL/lpr mice; comparison of results from the vehicle and combination treatment groups. **(C)** FACS of caspase-1/PI double positive cells in peripheral blood from MRL/lpr mice who were given or not given combination treatment. **(D)** Quantification of caspase-1/PI double positive cells in peripheral blood of MRL/lpr mice given or not given combination therapy. **(E)** Quantification of serum IL-1β concentrations in MRL/lpr mice given or not given combination treatment. ^####^P < 0.0001. FACS, fluorescence-activated cell sorting; CT, combination therapy.

Next, we examined caspase-1 and GSDMD expression levels in combination therapy and vehicle control groups. Immunohistochemical analysis revealed that the glomerular and renal interstitial caspase-1 and GSDMD expression was markedly decreased in the combination therapy group **(** **Figure 3A** **)**. Immunoblotting analysis also found a decreased expression and cleavage of caspase-1 and GSDMD in the kidney cortices of the combination treatment group **(** **Figure 3B** **)**. Moreover, caspase-1/propidium iodide (PI) double positive cells were counted using flow cytometry and a pyroptosis FAM Caspase-1 kit. The results revealed a marked reduction in caspase-1^+^/PI^+^ peripheral blood cell numbers in MRL/lpr mice that received the combination treatment in comparison with MRL/lpr mice treated with vehicle. **(** **Figures 3C, D** **)**. In addition, combination treatment also significantly reduced the serum IL1β concentration **(** **Figure 3E** **)**. We thus concluded that combination treatment suppressed caspase-1/GSDMD-induced pyroptosis in the kidneys and blood cells of LN mice.

### Combination Therapy Suppressed Caspase-1/GSDMD-Induced Pyroptosis in LN Patients

**Figure 4:**
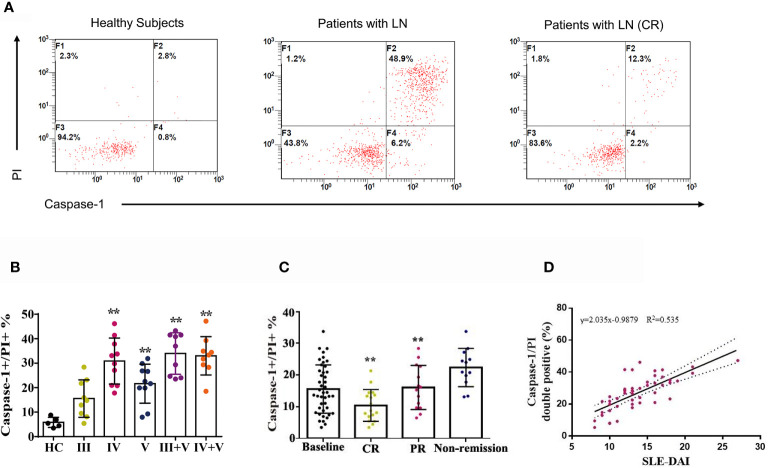
Combination therapy attenuated caspase-1-mediated pyroptosis in LN patients. **(A)** FACS of caspase-1/PI double positive cells in peripheral blood of LN patients treated with or without combination therapy. **(B)** Quantification of caspase-1/PI double positive cells in peripheral blood of type III, IV, V, III+V and IV+V LN patients and heathy subjects. **(C)** Quantification of caspase-1/PI double positive cells in peripheral blood of LN patients at baseline, CR, PR and non-remission after combination therapy. **(D)** Correlation analysis of caspase-1/PI double positive cells ratios with SLE-DAI. ^**^ *p* < 0.01. FACS, fluorescence-activated cell sorting; LN, lupus nephritis; CR, complete remission; PR, partial remission; SLE-DAI, systemic lupus erythematosus disease activity index.

Between January 2020 and June 2020, 47 patients with LN were enrolled and 43 completed 24-weeks of combination therapy. The baseline disease and demographics of the enrolled cohort are listed in **Supplementary Table 1**. Quantification of caspase-1/propidium iodide (PI) double positive cells was conducted in patients with LN type III, IV, V, III + V, IV + V and in heathy controls using a pyroptosis FAM Caspase-1 kit and flow cytometry. Compared to the healthy controls, the numbers of caspase-1/PI double positive cells in peripheral blood were increased **(** **Figures 4A, B** **)**. We also examined caspase-1/PI double positive cells after 24-weeks therapy and at baseline. After 24-weeks of induction therapy, the incidence of CR and PR was 39.5% (17/43) and 32.6% (14/43), respectively. The caspase-1/PI double positive cell numbers in peripheral blood of CR and PR patients was remarkably reduced compared to baseline **(** **Figure 4C** **)**. We also identified a correlation between caspase-1 induced pyroptosis and the SLEDAI; the data clearly showed that the caspase-1/PI positive ratio in peripheral blood was positively correlated with SLEDAI **(** **Figure 4D** **)**.

### Ac-FLTD-CMK, a GSDMD-Derived Inhibitor, Prevented the Development of LN

**Figure 5:**
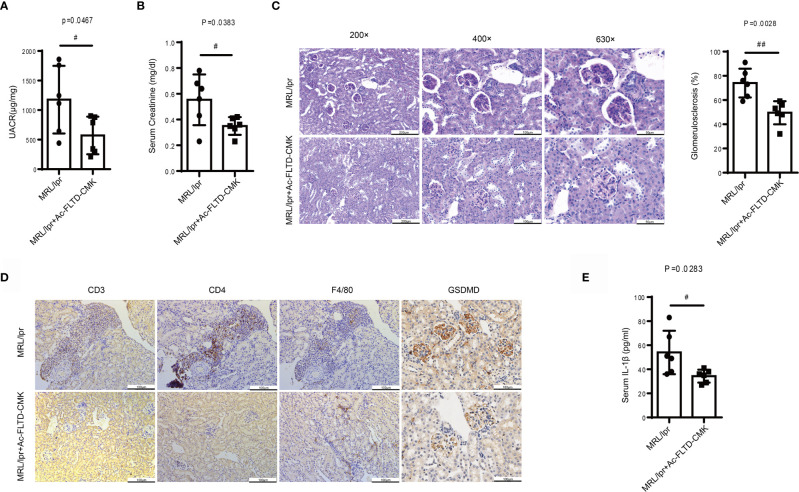
Ac-FLTD-CMK, a GSDMD-derived inhibitor, prevented the development of LN. **(A)** The urine albumin-to-creatinine ratio was calculated in MRL/lpr mice given or not given a GSDMD inhibitor. **(B)** Serum creatinine in MRL/lpr mice given or not given a GSDMD inhibitor. **(C)** Representative images of kidney specimens stained with Periodic acid–Schiff (PAS) and quantification glomerulosclerosis percentage. **(D)** Immunochemical staining of CD3, CD4, F4/80 and GSDMD. **(E)** Quantification of serum IL-1β concentrations in MRL/lpr mice given or not given a GSDMD inhibitor. Data are shown as the mean ± SEM. ^#^ *p* < 0.05, ^##^ *p* < 0.01.

A recent study found that GSDMD^-/-^ mice developed increased renal C3 and IgG deposition, more severe renal injury and enhanced mortality in an imiquimod-induced model of SLE.**^17^** To clarify further the role of GSDMD in LN, MRL/lpr mice were treated with Ac-FLTD-CMK**^21^** starting at 8-weeks-old, with vehicle as the control. After 8-weeks treatment, no difference was found in mortality or body weight between the vehicle and Ac-FLTD-CMK treated group. Compared to the vehicle control mice, Ac-FLTD-CMK-treated MRL/lpr mice had a lower urine albumin-creatinine ratio **(** **Figure 5A** **)**, a lower serum creatinine concentration **(** **Figure 5B** **)**, reduced glomerulosclerosis **(** **Figure 5C** **)** and CD3, CD4 and CD68 positive cell infiltration **(** **Figure 5D** **)**. Immunohistochemistry results showed a reduced expression of GSDMD **(** **Figure 5D** **)** and suppressed serum IL-1β secretion determined by ELISA **(** **Figure 5E** **)**. Collectively, the data suggested that Ac-FLTD-CMK inhibited pyroptosis in kidneys affected by LN.

## Discussion

Combination therapy is a promising strategy because of higher CR and overall response rates, a briefer time to remission and fewer side effects. Until now, the underlying molecular mechanisms of combination therapy were not known. Recently, Fu et al. (6) treated MRL/lpr mice with monotherapies or combination therapy and carried out a comprehensive transcriptomic analysis on whole kidneys. Interestingly, they found that combination therapy but not monotherapies specifically and uniquely suppressed caspase-1 expression.

The NLRP3/ASC/caspase-1 inflammasome is now recognized as a key contributor to the pathogenesis of LN. NLRP3 inflammasomes in podocytes are activated in lupus-prone mice and also in patients with LN. When NLRP3 inflammasomes were suppressed with a caspase-1 inhibitor, proteinuria, renal histological lesions and podocyte foot process effacement was found to be ameliorated in lupus-prone mice (8). Huang et al. (27) reported that NLRP3 was expressed in tubular cells of LN class IV and that NLRP3 activation was positively correlated with the activity index score for patients with LN. Caspase-1 has been proven to play a vital role in lupus and vascular dysfunction, and thus is a potential target for novel therapeutic interventions (28). Emerging experimental drugs such as RIP3 (29), P2X7 receptor antagonists (30), GSK-3β inhibitors (31) and an Nrf2 agonist (32) inhibited the development of LN in MRL/lpr mice by modulating he NLRP3/ASC/caspase-1 inflammasome activity, which highlights the importance and criticality of the NLRP3/ASC/caspase-1 inflammasome in LN. Raised serum concentrations of IL-18 have been shown to be correlated with disease severity and the degree of kidney injury/involvement in SLE patients (33). To the best of our knowledge, it seems that the gasdermin family might be essential for the development of pyroptosis, but there has been a paucity of evidence for the presence of GSDMD/GSDME in SLE patients.

Overall, we examined the activation and expression of GSDMD in kidney biopsy specimens taken from LN patients and MRL/lpr mice. GSDMD was strongly induced and cleaved in glomerular podocytes and tubular cells and infiltrated interstitial cells. Subsequently, MRL/lpr mice were given a combination of MMF, tacrolimus and prednisone or vehicle. Compared with the vehicle control group, activation of caspase-1 and GSDMD was reduced in the combination treatment group. The degree of pyroptosis in peripheral blood was also much lower in LN patients after administration of combination therapy. Moreover, pyroptosis in peripheral blood was positively correlated with SLEDAI, which suggested that pyroptosis in peripheral blood could act as a significant marker for SLE activity. We thus hypothesized that combination therapy might confer beneficial effects in LN by inhibiting pyroptosis induced by caspase-1/GSDMD. Previous crystal structural studies of caspase-1 in a complex with Ac-FLTD-CMK revealed many enzyme-inhibitor interactions that prevented GSDMD recognition by caspase-1. However, we found that Ac-FLTD-CMK, which acts as a GSDMD-derived inhibitor, inhibited pyroptosis in LN, which might be induced by a different mechanism.

Recently, in a TLR7-induced murine model of SLE (34), GSDMD-/- mice developed a greater degree of kidney damage and increased mortality, which initially suggested that GSDMD deletion would have a protective effect and be associated with systemic inflammation and production of autoantibodies with associated increased mortality. GSDMD-deficient mice develop a more prominent deposition of a glomerular immune complex in kidney specimens and higher proteinuria compared with wild-type mice. Enhanced secretion of extracellular HMGB1 was also found in GSDMD-/lymphocytes suggesting that necrosis/pyroptosis still occurred when GSDMD was absent.

Caspase-1 triggers pyroptosis by GSDMD cleavage. In contrast, in GSDMD-deficient or GSDMD-low cells, caspase-1 triggers apoptosis and then GSDME-dependent secondary necrosis and pyroptosis. When GSDMD activity is absent after therapy, GSDME could perhaps also switch caspase-3-mediated apoptosis to pyroptosis (35, 36). In addition, the activation of caspase-8 was much enhanced in macrophages that were GSDMD deficient. Significant activation of caspase-8 was also found in TLR7-induced GSDMD-deficient LN mice (34). Thus, in the absence of GSDMD, it is likely that caspase-1 cooperates with caspase-8 to cause caspase-3-GSDME-mediated apoptosis to switch to pyroptosis in LN, a hypothesis that demands further experimental investigation.

There are several limitations in the current study. First, based on the current data, we verified that combination therapy could suppress pyroptosis, but other treatment such as dual therapy of steroids and MMF, steroids and tacrolimus or anti-CD20 antibody might also have comparable effects. Second, the exact molecular mechanism of inhibition effect of combination therapy on caspase-1 is not well be explored. Last but not least, we observe the relationship of caspase-1/PI double positive cells in peripheral blood with SLE-DAI, and the change before and after combination therapy treatment in a retrospective LN patient cohort. The clinical predictive value of caspase-1/PI double positive cells for diseases activity and treatment response need to be further investigated in a larger prospective cohort.

Overall, we identified that combination therapy conferred its beneficial effects by suppressing caspase-1/GSDMD-induced pyroptosis in LN, which provided a novel insight into the cellular mechanisms involved. Indeed, combination therapy suppressed caspase-1 expression specifically and uniquely. Pharmacological or genetic inhibition of NLRP3 and caspase-1 improved renal function and suppressed autoimmunity in LN. The pathophysiological role of caspase-1 in LN is worthy of further study, and therapies targeting GSDMD in patients with LN needs to be very carefully evaluated.

## Data Availability Statement

The raw data supporting the conclusions of this article will be made available by the authors, without undue reservation.

## Ethics Statement

The studies involving human participants were reviewed and approved by Institutional Review Board of the First Affiliated Hospital of Zhejiang University. The patients/participants provided their written informed consent to participate in this study. The animal study was reviewed and approved by Institutional Animal Care and Use Committee of the First Affiliated Hospital of Zhejiang University.

## Author Contributions

JLin and SJ: Study Design, Methodology. HC: Data analysis, Writing and Editing. JYL and JLiu: Vivo and vitro experiment. YH and YK: Visualization, Investigation. YS: Software, Validation. All authors contributed to the article and approved the submitted version.

## Funding

This research was supported by the National Natural Science Foundation of China (81701602), National Key R&D Program of China (2020YFA0710800) and Social Development Grant of Jiangsu Province (BE2019720).

## Conflict of Interest

The authors declare that the research was conducted in the absence of any commercial or financial relationships that could be construed as a potential conflict of interest.

## Publisher’s Note

All claims expressed in this article are solely those of the authors and do not necessarily represent those of their affiliated organizations, or those of the publisher, the editors and the reviewers. Any product that may be evaluated in this article, or claim that may be made by its manufacturer, is not guaranteed or endorsed by the publisher.

## Supplementary Material

Supplementary Figure 1 Combination therapy suppressed proteinuria, glomerulosclerosis and renal interstitial immune cells infiltration in MRL/lpr.
