# Supplementary figures and images for: Novel Effects of Combination Therapy Through Inhibition of Caspase-1/Gasdermin D Induced-Pyroptosis in Lupus Nephritis
Source: Front Immunol. 2021 Nov 19;12:720877. doi: 10.3389/fimmu.2021.720877 (PMC8639704; doi:10.3389/fimmu.2021.720877)

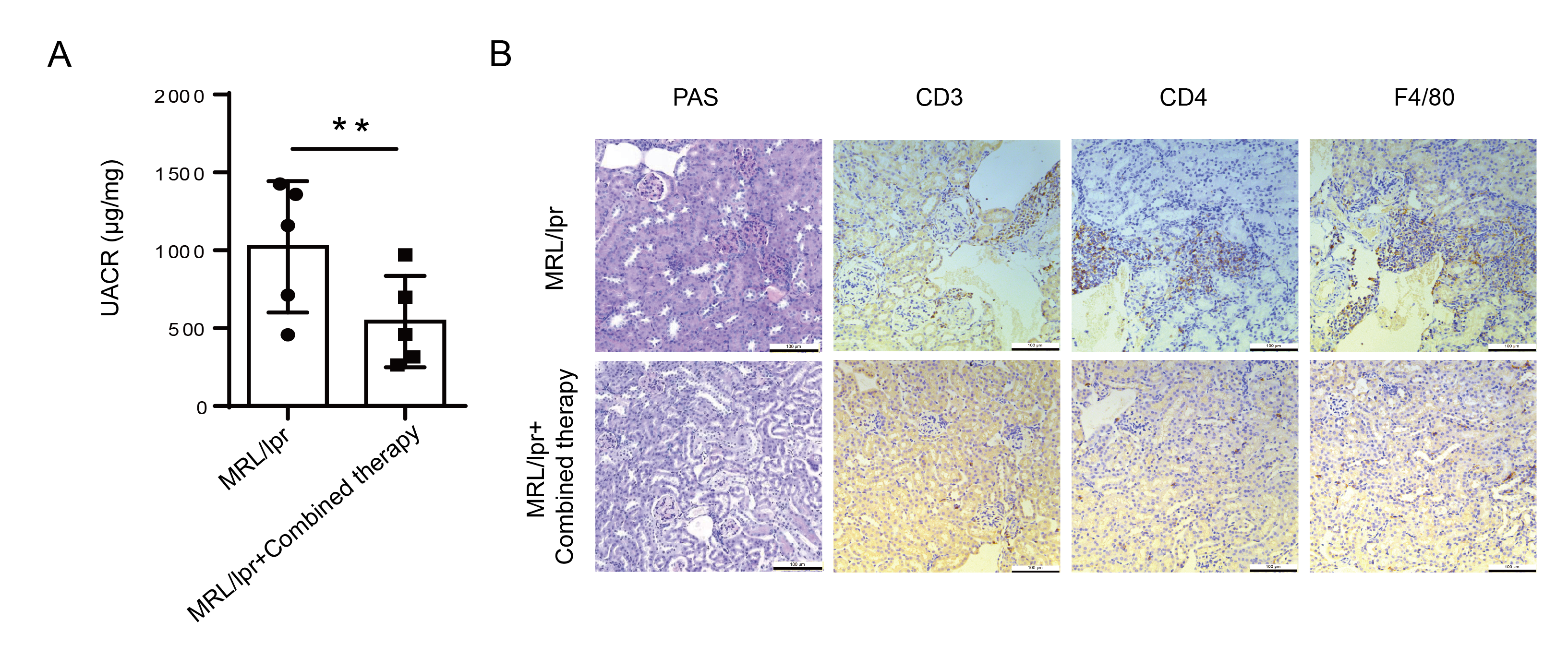

Supplement: Supplementary Figure 1 — Combination therapy suppressed proteinuria, glomerulosclerosis and renal interstitial immune cells infiltration in MRL/lpr. [file Image_1.tif]
